# Intraspecific Scaling of the Resting and Maximum Metabolic Rates of the Crucian Carp (*Carassius auratus*)

**DOI:** 10.1371/journal.pone.0082837

**Published:** 2013-12-20

**Authors:** Qingda Huang, Yurong Zhang, Shuting Liu, Wen Wang, Yiping Luo

**Affiliations:** Key Laboratory of Freshwater Fish Reproduction and Development (Ministry of Education), School of Life Science, Southwest University, Chongqing, China; Universität Bielefeld, Germany

## Abstract

The question of how the scaling of metabolic rate with body mass (M) is achieved in animals is unresolved. Here, we tested the cell metabolism hypothesis and the organ size hypothesis by assessing the mass scaling of the resting metabolic rate (RMR), maximum metabolic rate (MMR), erythrocyte size, and the masses of metabolically active organs in the crucian carp (*Carassius auratus*). The M of the crucian carp ranged from 4.5 to 323.9 g, representing an approximately 72-fold difference. The RMR and MMR increased with M according to the allometric equations *RMR* = 0.212*M* ^0.776^ and *MMR* = 0.753*M* ^0.785^. The scaling exponents for RMR (*b*_r_) and MMR (*b*_m_) obtained in crucian carp were close to each other. Thus, the factorial aerobic scope remained almost constant with increasing M. Although erythrocyte size was negatively correlated with both mass-specific RMR and absolute RMR adjusted to M, it and all other hematological parameters showed no significant relationship with M. These data demonstrate that the cell metabolism hypothesis does not describe metabolic scaling in the crucian carp, suggesting that erythrocyte size may not represent the general size of other cell types in this fish and the metabolic activity of cells may decrease as fish grows. The mass scaling exponents of active organs was lower than 1 while that of inactive organs was greater than 1, which suggests that the mass scaling of the RMR can be partly due to variance in the proportion of active/inactive organs in crucian carp. Furthermore, our results provide additional evidence supporting the correlation between locomotor capacity and metabolic scaling.

## Introduction

Body mass (M) is an important factor affecting the metabolic rates of animals. The metabolic rate can be scaled based on M according to the allometric equation 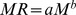, where MR is the metabolic rate; M is body mass; *a* is a constant; and *b* is the scaling exponent. Although many studies have focused on metabolic scaling and developed many significant theories [1]–[5], the *b*-value of scaling remains controversial [6]–[9]. One of the most debated scaling models is the metabolic theory of ecology, which assumes that resources are transported through fractal distribution networks in all organisms, and energy costs in these networks are minimized through evolution [1], [10]–[12]. According to the metabolic theory of ecology, a 0.75 power of M is universal for inter- and intraspecific metabolic scaling [1], [11]. However, the 0.75 scaling law has not been supported by many intraspecific studies, especially in fish. The *b*-value for the resting metabolic rate (RMR) (*b*_r_) of fish varies within a wide range, between 0.38 and 1.29 (mostly between 0.66 and 1), depending on taxonomic affinities, species lifestyle, and water temperature [8], [13]–[14]. The *b*_r_ of many fish species also varies during different ontogenetic phases [8], [15]–[16]. The RMR of some fish tends to increase nearly isometrically for larvae and early juveniles, then increases allometrically for large juveniles and adults [15], [17].

A dynamic energy budget (DEB) theory assumes that assimilation rate is proportional to the surface area of the structural body, while maintenance rate is proportional to the structural body volume [18]–[20]. The DEB theory predicts that the metabolic rate scaling exponent should vary between 0.66 and 1, depending on the relative proportions of the metabolically inactive ‘reserve’ and metabolically active ‘structure’ of different individuals [19], [21]–[22]. Unfortunately, most DEB parameters cannot be measured directly [21]. Similarly, the allometric scaling of the metabolic rate of animals has been explained by the organ size hypothesis, which states that organs with low metabolic rates become larger in weight in proportion to the whole body with increasing M [23], which is supported by the studies in the carp (*Cyprinus carpio*) and the porgy (*Pagrus major*) [24]–[26]. Accordingly, the mass scaling exponents of metabolically active organ sizes (heart, gill, etc.) have been reported to be less than 1 in cownose rays (*Rhinoptera bonasus*), Atlantic stingrays (*Dasyatis sabina*) [27], and largemouth bronze gudgeon (*Coreius guichenoti*) [28], whereas the mass scaling exponent of metabolically active organ size was found to be not significantly different from 1 in the brown tout (*Salmo trutta*) [29]. It appears that decreases in the size of metabolically active organs as M increases may not hold for all species of fish. Therefore, further studies in other fish species are required to test whether the various proportions of metabolically active and inactive organs contribute to the scaling of the metabolic rate.

Another important scaling model is provided by the metabolic-level boundaries (MLB) hypothesis, which predicts that volume and surface area constraints (scaling as M^1^ and M^2/3^, respectively) act as boundary limits on *b*, and the metabolic level (L) determines the relative importance of these constraints [3]–[4], [30]–[31]. According to the MLB hypothesis, the scaling slope for the RMR is inversely related to metabolic level [3], [30]. The MLB hypothesis has recently been supported by the negative relationship observed between the intraspecific scaling of the RMR and metabolic levels in 89 species of teleost fish [14]. In addition, according to the MLB hypothesis, the scaling exponent *b* (*b*_m_) for the maximum metabolic rate (MMR) of many species approximately equals 1.0, as has been shown in previous studies [3]–[4], [6]–[7], [9]. The MLB hypothesis has explained it as arising from muscular energy expenditure dominating an animal’s metabolism during maximal exercise, such that the scaling of muscle mass is directly proportional to *M* ^1^ [3]–[4], [6]. However, *b*_m_ values far from 1.0 have also been observed in some species [4], [7], [32]. It has been suggested that further experimental analysis is required to study the intraspecific scaling exponents in species that span a wide range of M [33]–[34].

A cell metabolism hypothesis has been proposed stating that the variation in metabolic scaling could be attributed to differences in cell size. Larger cells have relatively lower metabolic rate due to their relatively smaller surface area/volume ratio [35]–[36]. Therefore, scaling exponent *b* should be equal to 1.0 if an increase in body size is entirely due to an increase in cell number, or 0.66 if an increase in body size is entirely attributed to an increase in cell size [3], [35]. The cell metabolism hypothesis has been supported by studies addressing interspecific metabolic scaling in mammals, birds, and reptiles [36]–[37] and intraspecific metabolic scaling in ants [38], crayfish [39], frogs [35], and eyelid geckos [40]. However, there are few data available for testing the cell metabolism hypothesis in fish. The only recent study that has shown a positive effect of erythrocyte size on metabolic rates was conducted in a hybrid species of fish (*Cobitis taenia*) [41]. It is unclear if this hypothesis is generally applicable to intraspecific metabolic scaling in fish.

In the present study, we selected the crucian carp, *Carassius auratus* (Linnaeus, 1758), as an experimental animal. The crucian carp is a highly adaptable species with a wide distribution around the world. Previous studies in this species have reported basic physiological data on its RMR, MMR, metabolic scope, locomotor capacity, digestive performance, and hematological parameters [42]–[45]. This fish exhibits a higher RMR, MMR, and metabolic scope than some closely related species [42]–[43]; however, its aerobic swimming performance is relatively poor [45]. The mass scaling of both the RMR and MMR of this species and its relationship with variations in cell or organ sizes remain unknown. It is also interesting to study how the relationships among RMR, MMR, and metabolic scope change as M increases. This study aimed to observe the mass scaling of both RMR and MMR, to assess the effects of cell size and metabolically active organ size on intraspecific metabolic scaling, and to calculate the changes in aerobic scope associated with increasing M.

## Materials and Methods

### Ethics Statement

All animal handling and experiments were conducted in accordance with the ethical requirements and recommendations for animal care of the Fisheries Science Institution of Southwest University, China (Protocol No. wang-2011-721-R1). Our project was also approved by the National Natural Science Foundation of China (No. 31000958).

### Experimental Animals

Healthy crucian carp were obtained from local fisheries in Chongqing, China, in October 2012 and acclimated in a rearing system for 2 weeks prior to the experiment in the fisheries laboratory at Southwest University, China. The temperature of the dechlorinated freshwater in the system was maintained at 25.0±1°C; the oxygen concentration was maintained above 7 mg·L^−1^; and ammonia-N was kept below 0.015 mg·L^−1^ during the experiment. The fish were fed a commercial diet (Fenghuang Diet Co., Ltd, Chengdu, China; chemical composition: protein [30.3%], fat [2.9%], and digestible carbohydrate [10.0%]) once daily at a rate of 1% of their M.

### Respirometer and Measurements of Oxygen Consumption

The metabolic rates (MRs) of individual fish were measured using a continuous flow respirometer with a design modified from that of Luo and Xie [46]. The M of the experimental fish ranged from 4.5 to 323.9 g. Chambers of different sizes (0.13 L, 0.52 L, 0.86 L, and 1.20 L) were used depending on the M of the experimental fish. Up to 14 fish were subjected to measurements at the same time, and 1 chamber without fish was used as a control for measuring the background oxygen consumption. The following formula was employed to calculate the MR (mg O_2_ per h per fish):
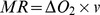
where Δ*O_2_* is the difference in the oxygen concentration (mg O_2_ per L) between the experimental chamber and the control chamber, and *v* (L·h^−1^) is the flow rate in the chamber.

The dissolved oxygen concentration was measured at the outlet of the chamber using an oximeter (HQ30d, Hach Company, Loveland, CO, USA). The flow rate of water through each respirometer chamber was measured by collecting water in a 100 mL beaker over a few minutes [47] and was adjusted to ensure a >7 mg·L^−1^ dissolved oxygen concentration in the outlet water to avoid stress on physiological processes [48]. The photoperiod was controlled using artificial lighting, with the light period covering 0800 to 2200 (14L: 10D), and the experimental temperature was maintained at 25.0±1°C.

### Experiment Protocol

#### Pre-exercise MR

The crucian carp were not fed for approximately 36 h before the experiment, which is a period that is sufficiently long to avoid post-prandial effects [42]. Then, the fish were placed in respirometer chambers individually and allowed to acclimate to the experimental conditions for 24 h. The MR was measured every hour from 0800 to 1100 on the next day. The mean MR obtained in the last 3 measurements was used as the RMR.

#### Post-exercise MR

Following measurement of the RMR, the fish were individually transferred from the respirometer to a chasing tank, where they were chased vigorously for 5 min to exercise them until exhaustion [43]. In a prior study, we found that 5 min of chasing could exhaust the fish [43], and no significant differences were found among fish chased for 5, 10, or 15 min [unpublished data]. After the exhausting exercise, the fish were immediately retransferred into their incipient respirometer chambers. Measurement of MR commenced 1 min later, which allowed sufficient time to exchange over 99% of the water inside the chamber. The MR of each individual was measured at 1 min intervals for the first 10 min post-exercise. Thereafter, the MR was measured at 15, 20, 30, 40, 60, 80, 100, 120, and 140 min. The measurements were continued in each individual until the MR had recovered to less than 120% of the RMR. The following metabolic parameters were calculated: (1) RMR (mg O_2_ per h per fish), the pre-exercise oxygen consumption rate; (2) MMR (mg O_2_ per h per fish), the peak post-exercise oxygen consumption rate; (3) factorial aerobic scope (FAS), calculated as the ratio of the MMR to the RMR; (4) excess post-exercise oxygen consumption (EPOC, mg O_2_ per fish), the excess MR above the RMR during the recovery process, calculated using the finite difference method; and (5) EPOC duration (h), the time from exercise to when the MR fell below 120% of the RMR in an individual fish.

#### Hematological parameters and organ masses

When the individuals had recovered from exercise, they were anesthetized using 0.15 g·L^−1^ tricaine methanesulfonate (MS-222). After M and body length (L) were measured, blood was collected from the fish via caudal artery puncture using a 1 mL syringe containing 0.04 g·L^−1^ anticoagulant (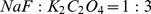). The hemoglobin (Hb) concentration was determined through the cyanmethemoglobin method [49] with a spectrophotometer (752, Modern Science Company, Shanghai, China) at a wavelength of 540 nm. Prior to measuring the absorbance, the samples were centrifuged (3,500×g for 5 min) to remove cell debris. The red blood cell count (RBCC) was determined using a Neubauer hemocytometer following prior dilution of the blood with 0.65% normal saline. Erythrocyte smears were produced using a Wright–Giemsa staining solution kit (including staining solutions A and B; Jinan Baboo Biotech Co., Ltd, Nanjing, China). The stained smears and the Neubauer hemocytometer were observed and photographed under a digital light microscope equipped with a video camera linked to a computer (Aigo Digital Technology Co., Ltd, Beijing, China). The length (LC) and width (WC) of 50 erythrocytes were measured for each fish. The erythrocyte surface area (S) of each individual was estimated using the equation 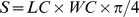 [50]. The amount of Hb per total surface area (Hb/TSAE) was calculated as 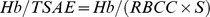 [44]. Because some individuals were too small for blood sampling, the final number of blood samples collected was 76.

Following blood sample collection, the experimental fish were killed. The heart, hepatopancreas, and the residual visceral organs (including digestive tract, spleen, gonads, kidneys), gills, and red muscle were then sequentially dissected out on ice and immediately weighed to the nearest 0.1 mg. The rest body was then stored at −80°C. The brain was sampled later from the unfrozen fish body and was also weighed. The total mass of active organs (M_Active organs_) was defined as the sum of heart, hepatopancreas, and the residual visceral organs (including digestive tract, spleen, gonads, kidneys), gills, brain, and red muscle. The total mass of active organs (M_Inactive organs_) was the difference between M and M_Active organs_.

### Statistics

The Data of this study are available from the Dryad Digital Repository at http://doi.org/10.5061/dryad.cr3p6. We declare that the data be freely available to any researchers if requested. The data were analyzed using SPSS 11.5 (SPSS Inc., Chicago, IL, USA). We did not analyze sexual differences in hematological parameters and metabolic rates because we were not able to determine the sex of the juvenile individuals. The crucian carp in our experiment, performed in October, were in reproduction phase II, which is a period during which only minor sex differences may exist. The fish were divided into 6 groups based on their M: <25 g, 25–50 g, 50–100 g, 100–150 g, 150–200 g, and >200 g to demonstrate the changes in metabolic rates before exercise and during recovery. The metabolic rate (mg O_2_ per h per fish) and M (g) were plotted on logarithmic axes. All data were log transformed prior to analysis. Estimates of the scaling exponent, *b*, were described with 95% confidence intervals (CIs). The correlations between metabolic rates and hematological parameters or organ masses were tested through partial correlations using M as the controlling variable and through linear regression analysis using residual values. The relationship between RMR and MMR was tested via linear regression analysis using residual values. The relationships between EPOC, hematological parameters, organ masses, and M were tested through linear regression analysis. Covariance analysis was performed to compare the difference between regression slopes. T-tests were used to compare the observed slope with the theoretical value of 0.75 or 1.0. Differences were considered significant when the *p* value was <0.05. Data are presented as the means ± standard error of the mean (SEM).

## Results

**Figure 1:**
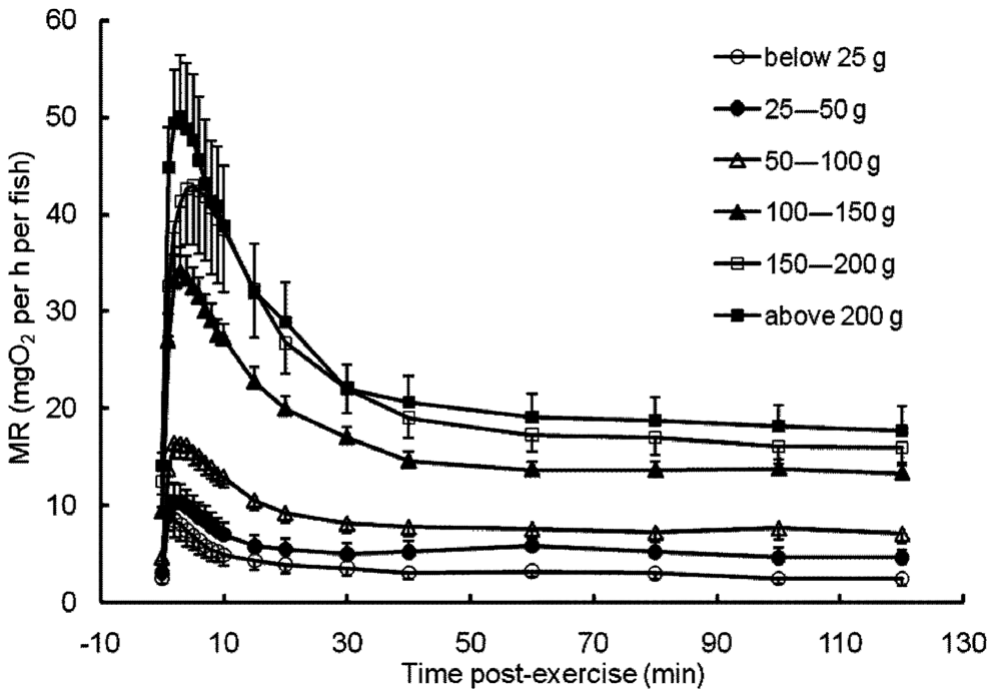
Metabolic rates (mg O_2_ per h per fish) of crucian carp before and after exercise. Open circle: less than 25 g; filled circle: 25–50 g; open triangle: 50–100 g; filled triangle: 100–150 g; open square: 150–200 g; filled square: greater than 200 g.

**Figure 2:**
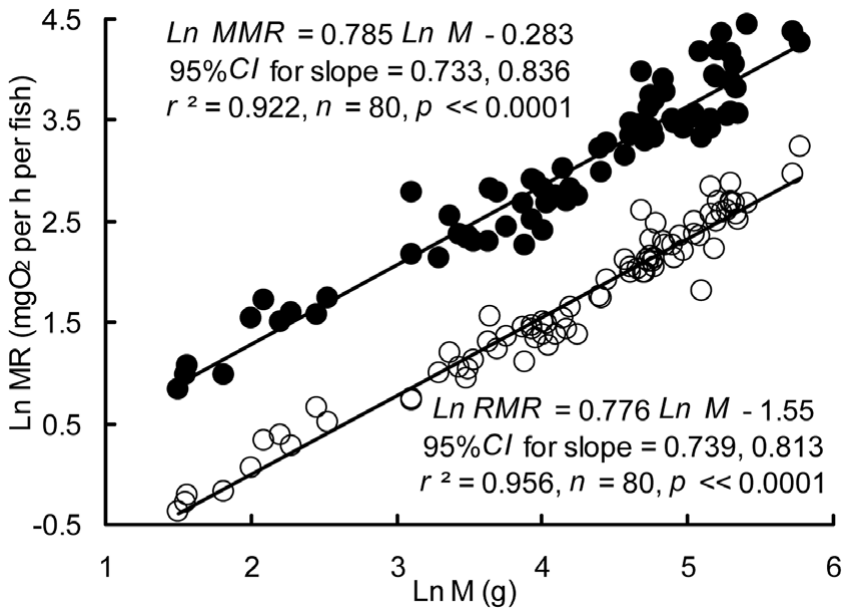
Metabolic rate (mg O_2_ per h per fish) versus body mass (g) in crucian carp. The filled circles represent the maximum metabolic rate (MMR; mg O_2_ per h per fish), and the open circles represent the resting metabolic rate (RMR; mg O_2_ per h per fish). The scaling exponent of the resting metabolic rate (*b*_r_) was 0.776 (standard error of the mean [SEM] = 0.019; 95% confidence interval [CI] = 0.739, 0.813), while the scaling exponent of the maximum metabolic rate (b_m_) was 0.785 (SEM = 0.026; 95% CI = 0.733, 0.836).

**Figure 3:**
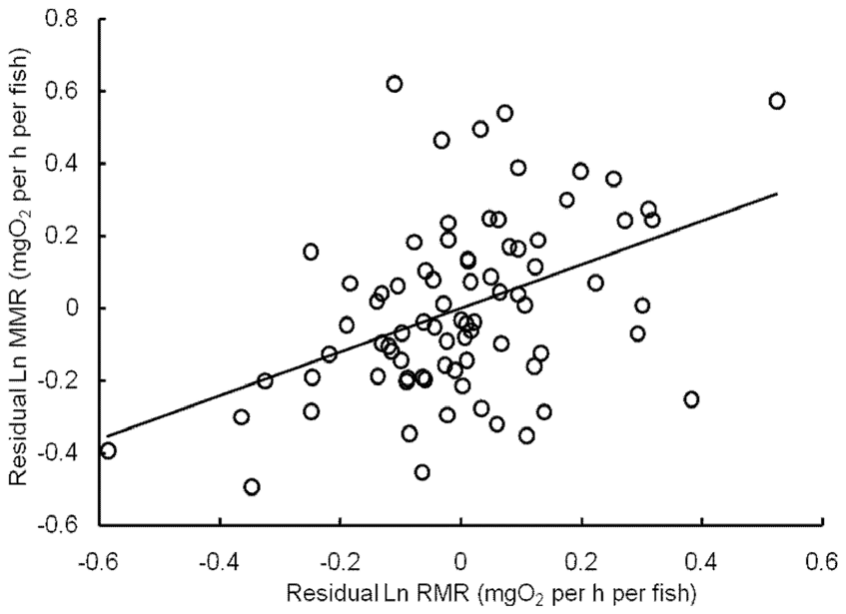
Relationship between the residual resting and maximum metabolic rates. RMR: resting metabolic rate (mg O_2_ per h per fish); MMR: maximum metabolic rate (mg O_2_ per h per fish).

**Figure 4:**
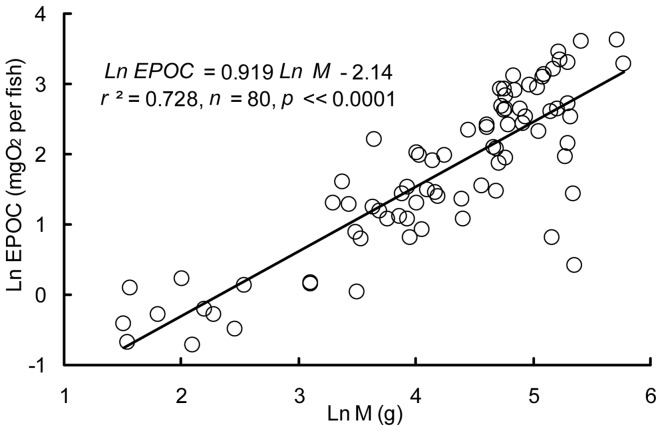
Relationship between excess post-exercise oxygen consumption (mg O_2_ per fish) and body mass (g).

The M of the experimental fish ranged from 4.5 to 323.9 g (*n* = 80), and the L ranged from 5.1 to 22.6 cm. The MRs of all individuals increased significantly at 2–5 min after the chasing exercise and then recovered slowly, reaching the pre-exercise value at approximately 100 min post-exercise (Fig. 1). The RMR of the crucian carp increased approximately 28 fold with increasing M, from 0.69 to 19.44 mg O_2_ per h per fish. The RMR increased with M by a scaling exponent of 0.776 (95% CI = 0.739, 0.813), which was not significantly different from 0.75 (*t = *1.385, *p = *0.170) (Fig. 2). Furthermore, the MMR increased nearly 37 fold with increasing M, from 2.30 to 85.94 mg O_2_ per h per fish. The MMR scaled with M by an exponent of 0.785 (95% CI = 0.733, 0.836), which also overlapped with 0.75 (*t = *1.351, *p = *0.181) but differed significantly from 1.0 (*t* = −8.300, *p*<<0.0001). The exponent *b*_r_ was not significantly lower than *b*_m_ (*F*_1, 158_ = 0.074, *p = *0.786), and the 95% CIs showed overlap. The average FAS of all individuals was approximately 4 and was almost constant with increasing M (Figs. 1 and 2). The RMR showed a positive relationship with the MMR after controlling for M (*r* ^2^ = 0.216; *p = *0.0000458) (Fig. 3). Additionally, the EPOC increased from 0.87 to 20.23 mg O_2_ per fish, with an exponent of 0.919 (*r* ^2^ = 0.728; *p*<<0.0001) (Fig. 4). However, the EPOC duration (h) did not change significantly with increasing M (*r* ^2^ = 0.026; *p = *0.156), and the mean recovery time of all individuals was approximately 100 min.

**Figure 5:**
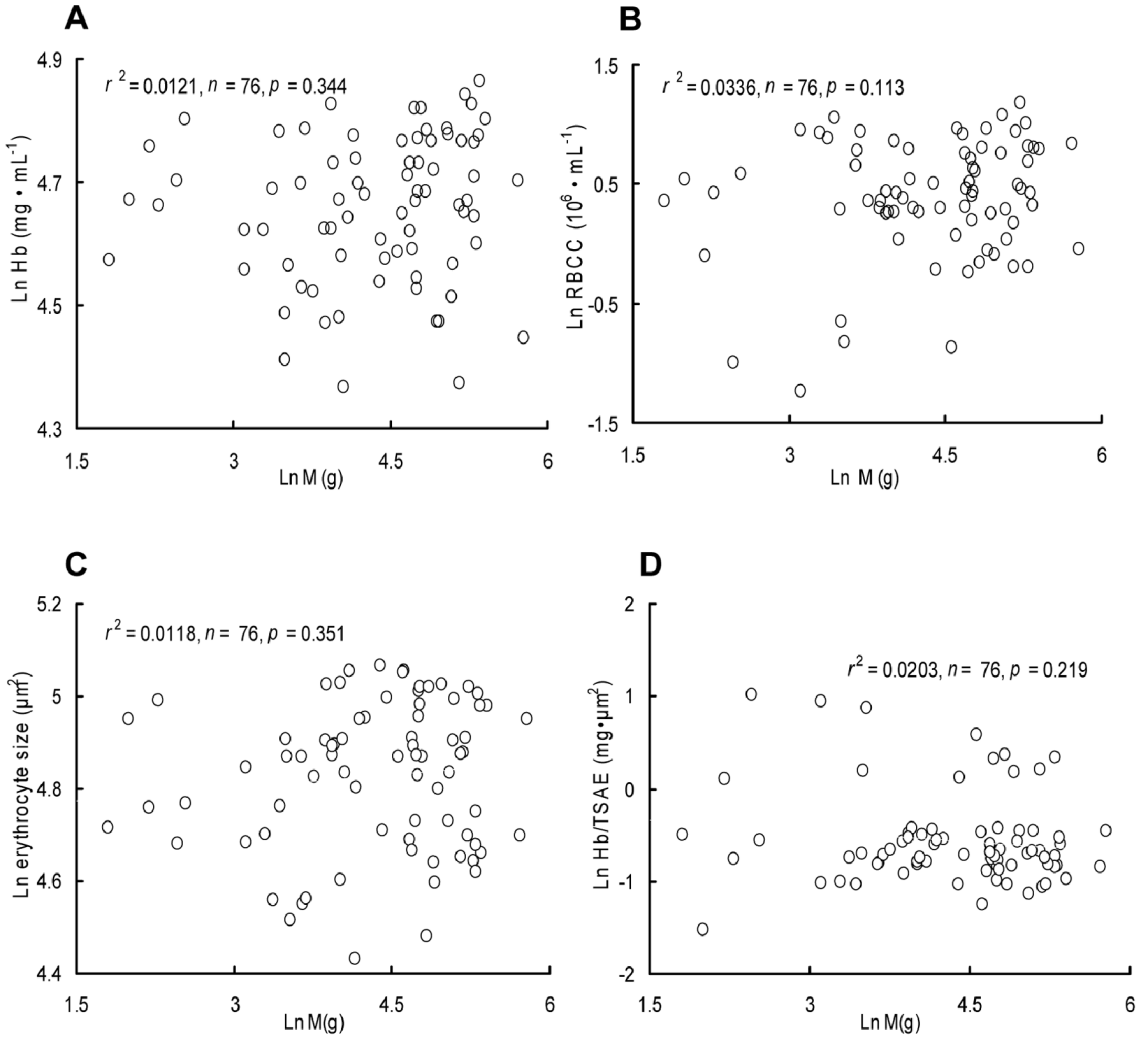
Relationships between hematological parameters and body mass (g). Hb: hemoglobin concentration (mg·mL^−1^); RBCC: red blood cell count (10^6^·mL^−1^); erythrocyte size (*µ*m^2^); Hb/TSAE: amount of Hb per unit surface area (ng·*µ*m^−2^).

None of the hematological parameters in our results were significantly correlated with M, including RBCC (*r* ^2^ = 0.034; *p = *0.133), erythrocyte size (*r* ^2^ = 0.012; *p = *0.351), Hb (*r* ^2^ = 0.012; *p = *0.344), and Hb/TSAE (*r* ^2^ = 0.020; *p = *0.219) (Fig. 5). Hb ranged from 78.7 to 129.6 mg·mL^−1^; RBCC from 0.29–3.26×10^6^ mL^−1^; erythrocyte size from 84.2–158.5 *µ*m^2^; and Hb/TSAE from 0.22–2.76 ng·*µ*m^−2^. No significant relationships were observed between the RMR and Hb (*r* ^2^ = 0.0437; *p = *0.074), RBCC (*r* ^2^ = 0.00539; *p = *0.534), or Hb/TSAE (*r* ^2^ = 0.00445; *p = *0.572) when controlling for M. However, absolute RMR was negatively correlated with erythrocyte size (*r* ^2^ = 0.0999; *p = *0.006) when controlling for M. Additionally, mass-specific RMR was also negatively correlated with erythrocyte size (*r* ^2^ = 0.086; *p = *0.011). No significant relationships were observed between the MMR and Hb (*r* ^2^ = 0.0378; *p = *0.097), erythrocyte size (*r* ^2^ = 0.00178; *p = *0.721), RBCC (*r* ^2^ = 0.0710; *n = *76; *p = *0.082), or Hb/TSAE (*r* ^2^ = 0.0512; *p = *0.053) when controlling for M.

**Figure 6:**
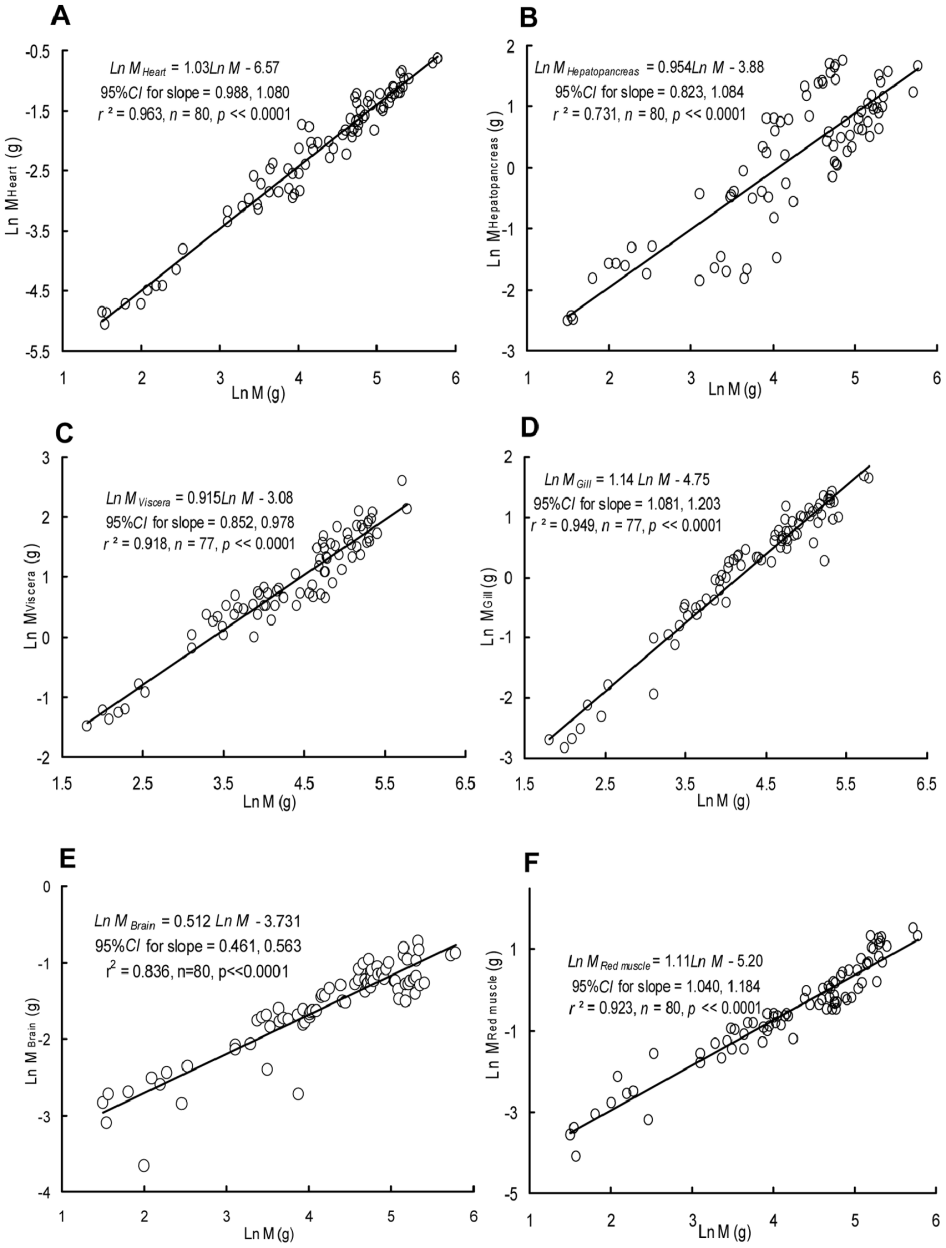
Relationships between the masses of different organs and body mass (g). The masses of the heart, hepatopancreas, residual visceral organs (including digestive tract, spleen, gonads, kidneys), gills, brain, and red muscle (g) are included.

**Figure 7:**
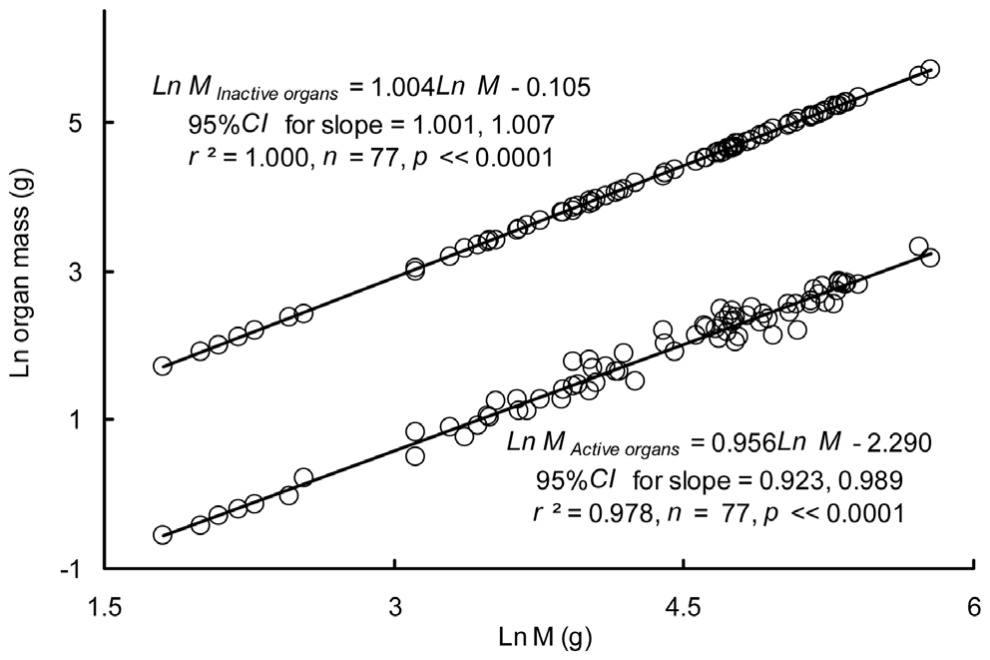
Relationships between the mass of active or inactive organs and body mass (g).

The masses of the heart (M_Heart_), hepatopancreas (M_Hepatopancreas_), residual visceral organs (M_Viscera_), gills (M_Gill_), brain (M_Brain_) and red muscle (M_Red_ _muscle_) were all positively correlated with M (Fig. 6). Both the *b* values for the heart (1.03; 95% CI = 0.988, 1.080) and the hepatopancreas (0.954; 95% CI = 0.823, 1.084) were not significantly different from 1.0 (M_Heart_: *t = *1.307, *p = *0.195; M_Hepatopancreas_: *t* = −0.702; *p = *0.485). The *b* values for M_Viscera_ (0.915; 95% CI = 0.852, 0.978) and M_Brain_ (0.512; 95% CI = 0.461, 0.563) were both below 1.0 (M_Viscera_: *t* = −2.685, *p = *0.00885; M_Brain_: *t* = −19.12, *p*<<0.0001), while both the *b* values for M_Gill_ (1.114; 95% CI = 1.081, 1.203) and M_Red_ _muscle_ (1.11; 95% CI = 1.040, 1.184) were greater than 1.0 (M_Gill_: *t = *4.567, *p = *0.0000176; M_Red_ _muscle_: *t = *3.039; *p = *0.00323). There were no significant correlations detected between the RMR and M_Heart_ (*r* ^2^ = 0.00007; *p = *0.941), M_Hepatopancreas_ (*r* ^2^ = 0.0016; *p = *0.725), M_Viscera_ (*r* ^2^ = 0.0123; *p = *0.337), M_Gill_ (*r* ^2^ = 0.0717; *p = *0.905), M_Brain_ (*r* ^2^ = 0.0008; *p = *0.805), or *M*_Red_ _muscle_ (*r* ^2^ = 0.0024; *p = *0.170) after controlling for M. The *b* value for the total mass of active organs (including the M_Red_ _muscle_, M_Gill_, M_Brain_, M_Heart_, M_Hepatopancreas_, and M_Viscera_) was 0.956 (95% CI = 0.923, 0.989), while the *b* value for the mass of inactive organs (the rest of the body) was 1.004 (95% CI = 1.001, 1.007) (Fig. 7), both of which were significantly different from 1.0 (M_Active organs_: *t* = −2.662, *p = *0.00475; M_Inactive organs_: *t = *2.622, *p = *0.00529). The proportion of M_Active organs_ was negatively correlated with M (*r* = −0.309, *p = *0.006) while M_Inactive organs_ was positively correlated with M (*r* = 0.309, *p = *0.006). There were no significant correlations between RMR and active organ mass (*r* = 0.0192, *p = *0.869) or inactive organ mass (*r* = 0.0191, *p = *0.870) after controlling for M.

## Discussion

The RMR of the crucian carp increased 28 fold as M increased 72 fold. Thus, the RMR scaled with M by an exponent of 0.776 (Fig. 2), which was close to the 0.75 scaling exponent suggested by the metabolic theory of ecology [1], [12], but fell within the range of values suggested by other theories described in the Introduction as well [3], [4], [19], [21], [35], [36]. Our scaling exponent was within the range of values reported for other teleost fishes (range, 0.38–1.29, mostly falling between 0.66–1) [8], [13]–[15]. Killen et al. [14] proposed an empirical relationship between the metabolic level (*L*) and scaling exponent (*b*): *b* = −0.145 Ln *L* +1.377, according to the MLB hypothesis [14]. Based on this empirical function, the logarithmic mass-specific RMR of crucian carp (4.54 mg O_2_ kg^−1^ h^−1^) at the midpoint of the regression (Ln M = 3.63 g) predicts a *b* of 0.718, which is only 7.5% deviated from the observed scaling exponent (0.776) for the RMR. Our results suggest that the function described by Killen et al. [14] can effectively predict the intraspecific scaling exponent of fish.

The cell metabolism hypothesis explains the mass scaling of metabolism by correlating cell size and body size [35]–[36]. This hypothesis predicts that larger cells have relatively lower metabolic rate, which is supported by the inverse correlations observed between erythrocyte size and RMR when controlling for M in one fish species (*C. taenia*) [41] and between erythrocyte size and mass-specific RMR in the grey partridge (*Perdix perdix*) [51]. Consistent with this hypothesis, erythrocyte size was found to be negatively correlated with either the absolute RMR when controlling for M or the mass-specific RMR in crucian carp. According to the cell metabolism hypothesis, the metabolic rate should increase nearly linearly with M in species that show invariant cell size [35], [36]. Recently, this hypothesis has been suggested to only hold true in vertebrates for erythrocyte size, rather than cell size in general, due to the role of erythrocytes in the oxygen supply [40]. It has been suggested that metabolic rate scaling is more linear in species with invariance in erythrocyte size during ontogeny, while an increase of erythrocyte size with body size during ontogeny should result in non-linearity in the relationship between metabolic rate and body size (i.e., flattening of metabolic rate scaling) [40]. We observed that the erythrocyte size of crucian carp did not change with increasing M (Fig. 5), whereas the RMR increased allometrically by an exponent of 0.776 (Fig. 2). There are several possible explanations for this finding. First, erythrocyte size may not represent the general size of other cell types in fish. Erythrocyte size is highly correlated with other cell types in different tissues in amphibians and birds [52], [53] and has been used to reflect general cell size [37], [41]; however, this relationship does not hold for mammals [53]. The correlation between the size of erythrocytes and other cell types requires further study in fish. Second, cell metabolism may change as M increases. In mammals, erythrocyte size is invariant with respect to body size, whereas the cellular metabolic rate is body size dependent [54], which may contribute to the scaling of metabolic rates. A decrease in the metabolic activity of cells, but not cell size, with increasing M has also been found in the hepatocytes of some other mammals [55]. Other factors, such as cell membrane permeability and the volume and activity of mitochondria, are body size dependent [28], [56]–[59], and these factors may contribute to metabolic scaling.

The masses of metabolically active organs have been suggested to be one of the factors contributing to metabolic scaling [23]. Studies conducted in carp and porgy have supported the hypothesis that allometric metabolic scaling can be partly attributed to a decrease in the relative mass of the active organs (e.g., heart, brain, hepatopancreas, kidney, digestive tract, red muscle, gills) in proportion to the whole body as fish grow [23], [25]–[26]. In the present study, the values of *b*_Heart_ (1.03) and *b*_Hepatopancreas_ (0.954) obtained for crucian carp showed nearly isometric increases associated with increasing M. Furthermore, the *b* values of the, gills and red muscle were higher than 1 (Fig. 6). However, the *b* values of brain and the residual visceral organs (including digestive tract, spleen, gonads, kidneys) were lower than 1. The total mass of active organs increased allometrically with M (b = 0.956), meaning that the proportion of active organ mass to M decreases throughout the ontogeny of crucian carp. On the other side, a *b* value of 1.004 for M_Inactive_ organs suggests that the proportion of inactive organ mass to M increases during ontogeny (Fig. 7). This finding suggests that the mass-scaling of the RMR can be partly attributable to variations in the proportion of active/inactive organs in crucian carp and indicates partial support the organ size theory by Oikawa et al [23], [25]–[26].

In many animals, the scaling pattern shifts during ontogeny, and the scaling exponent changes from near isometry during the larval phase to allometry during the juvenile phase and later, which may be correlated with a high energy cost of growth of larvae followed by an ontogenetic decrease in the growth cost [3], [60]–[62] and/or an ontogenetic decrease in the relative surface area of the respiratory organs [8], [15]–[17], [63]. Our results did not show a biphasic pattern for the metabolic scaling of the crucian carp. One potential explanation may be that the fish included in the present study were greater than 4.5 g and had passed the larval phase. Thus, the effect of the sharp ontogenetic switch in the growth cost was not observed in this study. Besides, although we did not measure the gill surface area directly, we can assume that the positive allometric growth pattern of gill mass (*b = *1.14) implies no biphasic allometric change from higher to lower relative gill surface area during the development stage of the crucian carp in this study, which could be an alternative reason for its constant scaling exponent of metabolic rate during ontogeny.

Interestingly, the scaling exponent for MMR (0.785) observed in the present study was quite low and was close to that for RMR (Fig. 2). The MMR scales with body size by an exponent of approximately 1.0 in many species of insects, fish, birds, and mammals [3]–[4], [6]–[7], [9]. This isometric scaling of the MMR has been attributed to metabolism being mainly influenced by volume-related muscle power production during exercise, which is determined by the mitochondria and capillary volume and scales as M^1^ [3]–[4], [6]. Nevertheless, *b*_m_ values do not significantly differ from *b*_r_ values in some ectothermic species [4], [32]. The low *b*_m_ value obtained for the crucian carp in our results suggests that the volume-related muscular energy expenditure has a smaller influence on the whole-body metabolic rate in this species. Consistent with this result, locomotor activity, or even strenuous exercise cannot exhaust the entire cardio-respiratory capacity of crucian carp [43]. It has been suggested that *b*_m_ values are related to a species’ lifestyle. The *b*_m_ value of athletic species is close to 1 because of the high mitochondrial content of their locomotor muscles and high volume of the capillary networks, whereas the *b*_m_ value of non-athletic species is low [7]–[8]. The crucian carp could be classified as a non-athletic species because it exhibits lower locomotor performance than most other cyprinid species [45], which would be consistent with its low *b*_m_ value. Our results support the correlation of locomotor capacity with metabolic scaling.

The homogeneous slopes of the increases in the MMR and RMR of the crucian carp with increasing M result in an almost constant FAS with increasing M (Fig. 2). Generally, large individuals display high FAS, implying a high potential metabolic capacity as their bodies grow [6], [8], [37], [64]. The invariant FAS of crucian carp suggests that the aerobic capacity might not increase as their bodies grow, which is consistent with its lower locomotor performance [45]. Accordingly, the Hb and Hb/TSAE of crucian carp were found to be similar, regardless of their M, indicating a constant oxygen transportation capacity (Fig. 5).

Our results also showed that crucian carp with a high RMR tended to exhibit a high MMR after controlling for M (Fig. 3), which is consistent with the results of many previous studies in vertebrates [29], [65]. This finding suggests that there is a cost of maintaining the machinery that supports the maximal aerobic capacity. On the other hand, our results showed that the EPOC of crucian carp increased with M (Fig. 4), which suggests that the anaerobic capacity of these fish increases during development [66].

In conclusion, in the present study, we quantified the allometric scaling of the RMR and MMR in the crucian carp. The obtained *b*_r_ and *b*_m_ values were close to each other, and the FAS was almost constant, which may be related with a non-athletic lifestyle. Our results also showed that the cell metabolism hypothesis could not explain metabolic scaling of the crucian carp and that erythrocyte size may not represent the general size of other cell types in this fish. More likely, the mass scaling of the RMR may be attributed in part to the variation in the proportion of the metabolically active organs. Finally, the positive relationship observed between the RMR and MMR supports the hypothesis that a high aerobic capacity is energetically costly to maintain.
